# The Effects of the MONOZUKURI Program on Executive Function Among Community‐Dwelling Older Adults: A Randomized Controlled Trial

**DOI:** 10.1002/brb3.70888

**Published:** 2025-09-27

**Authors:** Ai Iizuka, Susumu Ogawa, Daisuke Cho, Kyoko Fujihira, Yan Li, Kenichiro Sato, Keigo Hinakura, Kimi Estela Kobayashi‐Cuya, Hiroyuki Suzuki

**Affiliations:** ^1^ Research Team for Social Participation and Healthy Aging Tokyo Metropolitan Institute For Geriatrics and Gerontology Tokyo Japan; ^2^ Department of Psychology, College of Education Psychology and Human Studies, Aoyama Gakuin University Tokyo Japan; ^3^ Department of Liberal Arts Tokyo University of Technology Tokyo Japan

**Keywords:** cognitive function, cognitive intervention, creative activity, executive function

## Abstract

**Introduction:**

This study aimed to examine the effect of the MONOZUKURI program, a cognitive intervention related to creative activities (handicrafts and creative cooking), on cognitive function in community‐dwelling older adults.

**Methods:**

A single‐blind randomized controlled trial was implemented in Kanagawa, Japan, with examiners unaware of group allocations. Fifty‐one participants were randomly divided into two groups: the intervention group (IG; *n* = 29), which participated in 12 weekly sessions involving hands‐on activities such as crafting accessories and creatively preparing sushi; and the active control group (CG; *n* = 22), which attended health‐related educational lectures. Cognitive performance was evaluated both before and after the intervention. The main outcome measure was executive function, while secondary measures included verbal ability, attention, processing speed, memory, and working memory.

**Results:**

Using analysis of covariance, a significant group difference was detected in the change scores of the Trail Making Test Part B, which assesses executive function, indicating greater improvement in the IG. No statistically significant differences were found between groups in the other assessed cognitive domains.

**Conclusions:**

The MONOZUKURI program may have some effects on executive function among community‐dwelling older adults. Learning creative activity skills may contribute to the prevention of decline in executive function closely related to life functioning.

**Trial Registration:**

ClinicalTrials.gov identifier: UMIN000041679.

## Graphical abstract

This randomized controlled trial investigated the MONOZUKURI program's effects on cognitive function in older adults. The intervention group engaged in creative activities and showed greater improvement in executive function (TMT‐B) than the control group. Creative activities may help preserve executive function in community‐dwelling older adults.

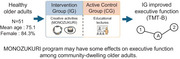

## 1 Introduction

Dementia prevention has become a critical public health concern (Livingston et al. 2020, 2017, 2024; Peters et al. 2019; Prince et al. 2015; World Health Organization 2021). Despite continued efforts to develop a fundamental cure for dementia, there has also been growing emphasis on preventive strategies, particularly those addressing modifiable lifestyle‐related risk factors (Alves et al. 2013; Livingston et al. 2020, 2017, 2024; Zülke et al. 2024; Yaffe et al. 2024; Winblad et al. 2016).

Cognitive reserve is considered to explain why some older adults maintain cognitive function despite substantial neuropathological changes (Y. Stern 2012, 2013; Amieva et al. 2014; Cholerton et al. 2016). This reserve can be partly enhanced through cognitively stimulating activities—such as education, leisure activities, and social engagement—which may support enriched neural networks (Stern et al. 2020; Valenzuela and Sachdev 2006). In this context, incorporating such elements into preventive interventions may represent a promising approach to dementia prevention.

Cognitive leisure activities (CLAs), defined as enjoyable and intellectually stimulating activities that promote well‐being, have the potential to enhance cognitive reserve (C. Stern and Munn 2019). These activities are often accessible even for individuals with physical limitations, as they are typically low‐impact and can be easily integrated into daily life. A notable strength of CLA‐based interventions lies in their practicality—they can be implemented in real‐world settings without substantial burden. Interventions that include skill acquisition, social interaction, and a high level of intellectual stimulation have been found to be particularly effective in promoting cognitive health (Iizuka et al. 2019a). In this study, we focused on “MONOZUKURI,” an activity that includes these common elements and is closely related to life functioning, to develop a program aimed at increasing cognitive reserve.

“MONOZUKURI” means “creating or manufacturing things” in Japanese, and the target productions are wide ranged, including crafts, ceramics, traditional crafts, and commercial products (Asai et al. 2011). Particularly, it is often used to describe the traditional Japanese elaborate and delicate work and product creation by skilled people. Creativity, which is at the core of MONOZUKURI, is linked to novelty and is expected to be particularly related to frontal function (Matsutani et al. 2013; Goldberg 2018). Additionally, performing tasks that involve precise and complex processes using the hands and fingers may be effective for cognitive functions, including executive function (Matsutani et al. 2013; Kobayashi et al. 2018a, 2018b).

Hence, in collaboration with Allabout Lifeworks Co.,Ltd (AALW), a company that sells correspondence learning materials for creative activity, we developed the “MONOZUKURI program” to provide novel and highly intellectually stimulating activities that are easy for the elderly to engage in such as handicrafts and creative cooking in a group setting. The program incorporates three key components known to support cognitive function: skill acquisition, social interaction, and a high level of intellectual stimulation. Additionally, the finished product can be displayed, worn, given as a gift, or eaten with friends, which can provide a sense of satisfaction and motivation to continue during and even after the program is finished. Although the effects of MONOZUKURI in elderly individuals have been examined from the perspective of psychological and social well‐being (Price and Tinker 2014; Liddle et al. 2013; Burt and Atkinson 2012; Pöllänen and Weissmann‐Hanski 2019; Tzanidaki and Reynolds 2011), to our knowledge, no studies have directly examined the intervention effects on cognitive function.

Therefore, this study aimed to examine the effects of the MONOZUKURI program on cognitive function in community‐dwelling older adults.

## 2 Materials and Methods

### 2.1 Study Design and Participants

This single‐blind randomized controlled trial was conducted in Kawasaki City, Kanagawa, Japan. The intervention and the assessments were carried out in three separate small‐group formats between October 2020 and July 2021, with approximately 10 participants assigned to each cohort. Recruitment was conducted through leaflet distribution and direct explanations provided at regional brain health events regularly organized by Kawasaki City. These events included light cognitive exercise sessions and a simple, tablet‐based cognitive screening tool with limited clinical relevance not used in the present study. Attendees comprised both health‐conscious older adults and individuals with subjective concerns about memory. While this recruitment method may have introduced some degree of selection bias, it allowed for the inclusion of participants with diverse levels of cognitive self‐awareness and motivation. Participants were eligible for inclusion if they were aged 60 years or older, were capable of independently performing activities of daily living, and reported subjective memory complaints. Individuals were excluded if they had significant visual or hearing impairments that affected daily functioning, or if physicians judged them unable to participate due to severe medical conditions, physical disability, dementia, or neuropsychological disorders.

All participants received an explanation of the study's objectives, procedures, and ethical considerations, and provided written informed consent prior to enrolment. A total of 61 individuals who satisfied the inclusion criteria and agreed to participate were randomly allocated to either the intervention group (IG; *n* = 30) or the active control group (CG; *n* = 31).

**FIGURE 1:**
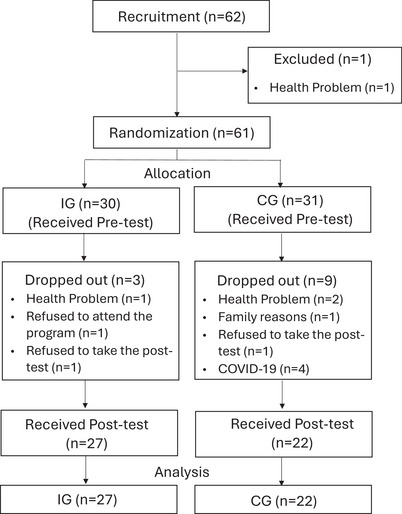
Study flow diagram following Consolidated Standards of Reporting Trials (CONSORT) guidelines.

As part of the study approval process, the research protocol and statistical analysis plan were reviewed and approved by the institutional review board and ethics committee of the Tokyo Metropolitan Institute for Geriatrics and Gerontology (TMIG). The trial was prospectively registered with the UMIN Clinical Trials Registry (ID: UMIN000041679), where a summary of the protocol is publicly accessible. The complete protocol and analysis plan can be obtained from the corresponding author upon request. A CONSORT (Hopewell et al. 2025) flow diagram is shown in Figure 1.

### 2.2 Intervention

#### 2.2.1 IG

The participants in the IG attended twelve 2‐h classes focused on learning handicrafts (making accessories) and creative cooking (decorating sushi), which were held once a week in the community center (Supporting Information). We modeled our program after the curriculum of AALW. The first six sessions focused on making accessary, with participants creating bracelets, pendants, and brooches. In the sixth session, the participants wore their finished products and were photographed. The latter six sessions centered on creative cooking, incorporating designs inspired by animals, seasonal events, and traditional Japanese patterns into the preparation of sushi. The materials were designed by instructors specializing in the production and teaching of handicrafts and creative cooking, and the level of difficulty was adjusted according to each participant's progress.

#### 2.2.2 CG

Participants in the active CG attended three 2‐h educational sessions held once a month, focusing on general health maintenance topics such as physical activity, nutrition, mental well‐being, and social engagement. These sessions were intentionally designed to be unrelated to the MONOZUKURI content, and served primarily to sustain participants’ engagement and adherence to the study protocol. They were not designed to provide cognitive or social stimulation equivalent to that of the IG.

The intervention included noninvasive, low‐risk activities such as handicrafts and creative cooking. The study was conducted during the COVID‐19 pandemic, and appropriate infection prevention measures—such as mask wearing, hand hygiene, physical distancing, and limiting group sizes—were implemented throughout all sessions. Although no systematic assessment of harms or adverse events was conducted, participants were encouraged to report any discomfort, allergic reactions, minor injuries, or symptoms suggestive of COVID‐19.

### 2.3 Measures

Outcome measures were specified for each domain of cognitive function. Executive function was designated as the primary outcome, based on its relevance to the complex cognitive demands involved in creative activities. Secondary outcomes included verbal ability attention, processing speed, memory, and working memory. Cognitive assessments were administered both prior to and following the 3‐month intervention period by examiners who were unaware of group assessments.

#### 2.3.1 Baseline Characteristics

Baseline participant characteristics included demographic information such as age, sex, and educational background, as well as medical history, self‐reported medication intake, and mental health status. Global cognitive functioning was assessed using two widely accepted tools: the Japanese version of the Mini‐Mental State Examination (MMSE‐J; Folstein et al. 1975; Sugishita and Hemmi 2010) and the Japanese adaptation of the Montreal Cognitive Assessment (MoCA‐J; Fujiwara et al. 2010). Each test is scored out of 30 points. The MMSE‐J is commonly employed as a screening tool for dementia, with a standard cutoff score of 23, while the MoCA‐J is considered more sensitive to detecting mild cognitive impairment, with a threshold of 25 points.

Mental health was evaluated using the Japanese version of the Geriatric Depression Scale, Short Form (GDS‐SF; Sheikh and Yesavage 1986; Sugishita and Asada 2009). This instrument consists of 15 items, with total scores ranging from 0 to 15. A score of 6 or higher was interpreted as indicative of depressive symptoms.

#### 2.3.2 Cognitive Assessments

##### 2.3.2.1 Executive Function

To evaluate executive function, we employed the Trail Making Test Part B (TMT‐B; Reitan 1958; Suzuki et al. 2022). This test involves 25 circles randomly arranged on a sheet of paper, each marked with either a number from 1 to 13 or one of the first 12 hiragana characters from the Japanese alphabet. Participants were instructed to connect the circles in alternating sequence—for example, “1” followed by “a” then “2” and so on—as quickly and accurately as possible. Faster completion times were interpreted as indicating better executive functioning.

##### 2.3.2.2 Verbal Ability

To assess verbal ability, we used two types of verbal fluency tasks: letter fluency and category fluency (Crossley, D'Arcy, and Rawson 1997; Shao et al. 2014). In the letter fluency task, participants were asked to generate as many words as possible in 1 min beginning with a given Japanese hiragana syllable (e.g., “ka” or “ho”). In the category fluency task, they listed items belonging to semantic categories such as animals or vegetables. The total number of responses from each pair of tasks was calculated, with higher scores reflecting better verbal ability.

##### 2.3.2.3 Attention

To assess attention, we used the Trail Making Test Part A (TMT‐A; Reitan 1958; Suzuki et al. 2022). In this task, participants were instructed to connect numbers in sequential order from 1 to 25 as quickly and accurately as possible. Faster completion times were interpreted as reflecting better attention.

##### 2.3.2.4 Processing Speed

To assess processing speed, we used the Coding subtest of the Wechsler Adult Intelligence Scale–Third Edition (WAIS‐III; Wechsler 1997). In this task, participants referred to a number–symbol key and filled in the corresponding symbols for each number as quickly and accurately as possible within a 2‐min time limit. The final score was the total number of correctly completed items, with higher scores reflecting better processing speed.

##### 2.3.2.5 Memory

To assess memory, we used the Logical Memory I and II subtests from the Wechsler Memory Scale–Revised (WMS‐R; Wechsler 1987). Participants were asked to recall the content of two short stories read aloud by the examiner. In the immediate recall task (Logical Memory I), recall was assessed immediately after the stories were presented. In the delayed recall task (Logical Memory II), participants were asked to recall the same stories after a 30‐min interval. Each subtest had a maximum score of 50, with higher scores reflecting better memory performance.

##### 2.3.2.6 Working Memory

Working memory was assessed using the Visual Memory Span Test (VMST) from the WMS‐R (Wechsler 1987) and the Digit Span Test (DST) from the WAIS‐III (Wechsler 1997). The VMST consists of forward (VMSF) and backward (VMSB) subtests. In the VMSF, the examiner touched sequences of squares printed on a test sheet, and participants were asked to repeat the sequences in the same order. In the VMSB, participants were asked to repeat the sequences in reverse order. The total VMST score ranged from 0 to 26, with higher scores indicating better visual workingmemory. The DST consists of forward (DSFT) and backward (DSBT) subtests. In the DSFT, participants were asked to memorize and repeat a sequence of numbers in the same order. In the DSBT, participants were asked to memorize the sequence and repeat it in reverse order. The total DDST score ranged from 0 to 28, with higher scores indicating better verbal working memory.

### 2.4 Sample Size

Based on previous studies involving interventions using CLAs, we anticipated a medium effect size for changes in the TMT‐B scores, which served as the primary outcome in this study. Sample size was calculated using G*Power (University of Düsseldorf, Düsseldorf, Germany), assuming 80% statistical power, a two‐sided test, a significance level of 0.05, and an analysis of covariance (ANCOVA) framework for between‐group comparisons over time. Accounting for an expected 15% attrition rate, the required sample size was estimated to be 104 participants.

However, due to national COVID‐19 safety guidelines that imposed restrictions on group activities, including limitations on interpersonal contact, the planned sample size was reduced by approximately half during recruitment to comply with pandemic‐related regulations.

### 2.5 Randomization

Participants were randomly assigned to either the IG or CG in a 1:1 ratio using simple randomization. Random numbers were generated via the RAND function in Microsoft Excel 2019 (Microsoft Corp., Redmond, WA, USA). Each participant was assigned a numeric code, and personal identifiers were removed during the allocation process. Randomization was conducted independently by a study coordinator who was not involved in recruitment or intervention procedures. Allocation concealment was maintained throughout the process. After group assignment, AALW. informed participants of their assigned group via postal mail.

### 2.6 Blinding

Due to the nature of the intervention, neither participants nor the intervention providers were blinded to group allocation. The researchers and data analysts were also not blinded. However, examiners who administered the cognitive tests were blinded to participants' group assignments.

### 2.7 Statistical Analysis

To assess the impact of the intervention on cognitive outcomes, change scores were calculated by subtracting pretest values from posttest results. These change scores served as the dependent variables in separate ANCOVAs conducted for each cognitive measure, with group assignment (IG vs. CG) as the independent variable.

Sex, age, educational attainment, and baseline scores were included as covariates to control for initial group differences. Effect sizes were expressed using ηp^2^. Statistical significance was determined using two‐tailed *p*‐values, with a threshold set at 0.05. Analyses were performed using SPSS version 23 (IBM Corp., Armonk, NY, USA).

Missing data were not imputed and were analyzed as missing. No interim analyses or stopping criteria were established, as the study involved a low‐risk intervention over a brief time period.

## 3 Results

### 3.1 Compliance With the Program

The participant characteristics for those who completed the posttest are shown in Table 1. In total, three individuals from the IG and nine from the CG withdrew from the study. In the IG, one participant was hospitalized due to a serious medical condition unrelated to the program, one declined further participation in the MONOZUKURI sessions, and one did not attend the posttest. In the CG, reasons for dropout included two hospitalizations, one family related withdrawal, two refusals to complete posttesting, and two withdrawals due to concerns about COVID‐19 infection. As a result, 27 participants in the IG and 22 in the CG were included in the final analysis.

**TABLE 1:** Demographic characteristics at baseline for the IG and CG.

		IG (*n* = 27)	CG (*n* = 22)
Age (mean ± SD)	Years	74.9 ± 5.5	75.4 ± 6.3
Sex (male/female)	*N*	4/23	4/18
Education (12/13 years)	*N*	12/15	8/14
GDS‐15 [≦5/6≦]	*N* [Score (0–15)]	23/4	16/6
MMSE‐J (mean ± SD)	Score (0–30)	28.1 ± 1.9	28.9 ± 1.2
MoCA‐J (mean ± SD)	Score (0–30)	23.6 ± 3.1	23.8 ± 3.8

Abbreviations: CG, control group; GDS‐15, 15‐item Geriatric Depression Scale; IG, intervention group; MMSE‐J, the Japanese version of the Mini‐Mental State Examination; MoCA‐J, the Japanese version of the Montreal Cognitive Assessment; *N*, number of participants; SD, standard deviation.

Attendance rates in the MONOZUKURI program averaged 94.9%, with all participants attending at least 70% of sessions. No adverse events, including COVID‐19 cases, were reported in the IG. In contrast, a few COVID‐19 infections were noted in the CG, although these did not coincide with the timing of any cognitive assessments.

### 3.2 Effects on Cognitive Function

The descriptive statistics for pre‐ and post‐intervention cognitive test scores are presented in Table 2. An independent samples *t*‐test was conducted to compare baseline scores between the IG and the CG, and no significant differences were observed.

**TABLE 2:** Cognitive test scores of participants who completed the pretest and posttest.

		IG (*n* = 27)			CG (*n* = 22)		*p*‐value^a^
		Pre	Post		Pre	Post	
Executive function							
TMT‐B	Seconds to completion	127.8 ± 70.8	116.5 ± 54.0		124.8 ± 47.1	160.0 ± 106.5	0.87
Verbal function							
Letter	Number of words	18.6 ± 5.7	20.7 ± 6.1		19.1 ± 5.3	19.0 ± 5.7	0.75
Category	Number of words	31.2 ± 7.3	32.2 ± 7.8		31.7 ± 7.9	28.4 ± 8.0	0.84
Attention							
TMT‐A	Seconds to completion	42.3 ± 16.3	38.3 ± 14.8		48.4 ± 16.7	51.2 ±32.0	0.20
Processing speed							
Coding	Score	66.4 ± 13.8	64.0 ± 17.2		62.0 ± 13.8	60.1 ± 14.0	0.26
Memory							
LM I	Score	21.6 ± 7.0	21.4 ± 6.6		20.2 ± 5.8	22.0 ± 6.8	0.45
LM II	Score	15.0 ± 8.0	16.7 ± 7.9		13.3 ± 7.7	16.4 ± 8.0	0.45
Working memory							
DST	Score	11.9 ±3.1	12.7 ± 3.7		11.4 ± 3.1	11.7 ± 3.5	0.59
VMST	Score	16.0 ± 3.3	16.0 ± 2.6		15.5 ± 2.8	14.9 ± 2.7	0.60

Abbreviations: Category, category fluency; CG, control group; DST, digit span test; IG, intervention group; Letter, letter fluency; LM, logical memory; *n*, number of participants; TMT, trail making test; VMST, visual memory span test

^a^ *p*‐values are from independent sample *t*‐tests.

To assess the effects of the intervention on cognitive performance, ANCOVAs were performed on the change scores for each cognitive measure, as shown in Table 3. For executive function, which was the primary outcome of this study, a significant group difference was identified in the change scores for the TMT‐B. The IG showed greater improvement compared with the CG (*F*(1, 48) = 5.09, *p* = 0.03).

**TABLE 3:** Cognitive test change scores of participants who completed the pretest and posttest.

		IG			CG					
		Mean ± SD	95%CI		Mean ± SD	95%CI		*F*‐value	*p*‐value^a^	Effect size (ηp^2^)
Executive function										
TMT‐B	Seconds to completion	−11.3 ± 54.2	−36.6 to 16.0		35.1 ± 79.2	4.6 to 63.0		5.09	0.03*	0.106
Verbal function										
Letter	Number of words	2.0 ± 5.6	−0.1 to 3.9		−0.2 ± 5.8	−2.2 to 2.3		1.49	0.23	0.03
Category	Number of words	0.9 ± 7.0	−1.8 to 3.2		−3.3 ± 8.4	−5.8 to −0.3		4.06	0.05	0.09
Attention										
TMT‐A	Seconds to completion	−4.0 ± 10.2	−13.2 to 3.8		2.9 ± 30.8	−5.7 to 13.1		1.75	0.19	0.04
Processing speed										
Coding	Score	−2.5 ± 11.4	−6.0 to 2.2		−1.8 ± 9.9	−7.1 to 2.1		0.04	0.85	0.00
Memory										
LM I	Score	−0.1 ± 5.6	−1.8 to 1.9		1.9 ± 4.9	−0.5 to 3.7		1.28	0.26	0.03
LM II	Score	1.7 ± 8.6	−0.5 to 4.6		3.1 ±6.4	−0.1 to 5.5		0.14	0.71	0.00
Working memory										
DST	Score	0.8 ± 2.7	−0.2 to 2.0		0.3 ± 3.3	−1.0 to 1.4		0.78	0.38	0.02
VMST	Score	−‐0.0 ± 2.4	−0.6 to 0.7		−0.6 ± 2.0	−1.5 to −0.0		2.92	0.10	0.06

Abbreviations: Category, category fluency; CG, control group; DST, digit span test; IG, intervention group; Letter, letter fluency; LM, logical memory; SD, standard deviation; TMT, trail making test; VMST, visual memory span test.

^a^ *p*‐values are from analysis of covariance, adjusted for sex, age, and educational level along with the pretest scores.

*p < 0.05.

No statistically significant differences were found between the groups in any of the other cognitive measures. Full results, including test scores and ANCOVA statistics, are provided in Table 3.

## 4 Discussion

This study aimed to examine the effects of the MONOZUKURI program on cognitive function in community‐dwelling older adults. The results revealed a significant intervention effect of the MONOZUKURI program on the primary outcome, the TMT‐B.

Executive function assessed by the TMT‐B is not a single, but a comprehensive function of planning, initiating, and maintaining tasks in accordance with an ultimate goal. Creative activities, such as handicraft and creative cooking, require a complex process of deciding on one's own design with the ultimate goal of completing the work, planning how to achieve the final form, and maintaining the work according to the rules that have been decided. Repeating this process during the program, coupled with exposure to various stimuli through the creation of different works in each session, might have influenced executive function. Moreover, previous studies reported an association between hand dexterity and executive function (Kobayashi et al. 2018a, 2018b), and the repetition of tasks involving elaborate and complex processes using the hands and fingers in the program might also have affected executive function.

As this study was conducted during the COVID‐19 pandemic, under restricted social interaction, we considered that having the opportunity to communicate with others during the program may have had a positive effect on verbal function. However, no significant intervention effect on such cognitive domain was observed in this study. Programs that showed intervention effects on verbal fluency focus on training, such as comprehensive reading and articulation trainings (Calatayud et al. 2023; Zimmermann et al. 2014). Also, programs that include more verbal elements than just normal conversation may be more effective in improving verbal function. However, although no statistically significant differences were obtained, the IG tended to maintain category fluency scores even during the COVID‐19 pandemic, in which opportunities for communication were less frequent than usual. The communication within this program might have played a defensive role against decline in verbal function.

In addition to verbal function, no significant intervention effects were observed in other cognitive functions probably because this program did not require sustained focus or speed of work. To communicate with the instructor and other participants during the work or to adjust the progress of the work at the pace of the participants are advantages of this program. However, to improve attention and processing speed, a time limit or a certain amount of work speed may be required. Further, this program had few elements, in which the participants memorize specific information or take next action while retaining novel information. Therefore, the stimulation might have been insufficient to produce intervention effects on memory and working memory. These results are logically reasonable as previous studies have shown that the effects on cognitive function may vary depending on the characteristics of the program or activity (Suzuki et al. 2014; Iizuka et al. 2019b).

In this study, a variety of cognitive domains were assessed, including executive function, memory, attention, processing speed, verbal fluency, and working memory. These outcome measures were selected based on the cognitive processes likely to be stimulated by the MONOZUKURI program, which requires participants to plan, adapt, and execute complex manual tasks while engaging in creative thinking. While a statistically significant improvement was observed only in executive function, this domain‐specific result may reflect the nature of the intervention, which emphasizes goal‐directed behavior, flexible thinking, and fine motor coordination. Future studies could further explore subtle cognitive changes in other domains by employing broader or more sensitive assessments and comparing different types of creative or hands‐on activities.

As aforementioned, this program revealed a potential benefit on executive function, and the high attendance rate and the small number of dropouts from the program suggest that this program can be conducted in the community setting as an evidence‐based, systematized program. In the IG of the present study, instructors adjusted the difficulty level of the tasks or selected tasks according to each participant's level of understanding. The ability to adjust the difficulty level of the task means that it could potentially accommodate people of different cognitive levels. Given the current emphasis on living well with dementia and dementia prevention (Ministry of Health, Labour and Welfare 2021), there is an increasing need for tools and programs that cater to people of all cognitive levels, promoting shared enjoyment. Thus, it is necessary to investigate the effectiveness of this program in terms of living well with dementia in the future.

## 5 Limitations

This study has several limitations that should be taken into account. First, although the sample size was calculated based on a medium effect size assumption, only 51 participants were included in the final analysis due to pandemic‐related restrictions. As such, the statistical power may have been insufficient to detect smaller but meaningful effects, particularly in secondary outcomes such as memory and attention. This limitation increases the likelihood of type II errors, and therefore, caution is needed when interpreting the generalizability of the results.

Second, the IG and CG differed not only in content but also in the degree of engagement and level of social interaction. While the MONOZUKURI program involved active participation in creative activities, the CG attended more passive health education lectures. Such differences may have influenced the intervention effects not only through cognitive stimulation, but also via increased participant motivation and interpersonal interaction. Future studies should increase the sample size and consider designing control conditions that are equivalent to the IG in terms of social and emotional stimulation.

Third, this study did not include long‐term follow‐up beyond the 3‐month intervention period. Since cognitive decline typically occurs over extended periods, the absence of long‐term follow‐up limits our ability to evaluate whether the observed effects are sustained over time. Although short‐term improvements were observed, the durability of these benefits remains unclear. Future longitudinal studies should examine the sustained effects of such interventions, considering both the frequency and duration of participation.

Fourth, the intervention activities, including handicrafts and creative cooking, may be more appealing to women. As a result, there was a sex imbalance among participants, which could have influenced the findings. Future studies should aim for more balanced gender representation. Recruitment strategies may also need to be adapted to better reach older men, such as by approaching retirement associations or senior men's clubs.

Finally, although outcome assessors were blinded, participants were necessarily aware of their group assignment due to the nature of the intervention. As in many lifestyle‐based trials, this may have introduced expectancy or placebo effects, which should be taken into account when interpreting the results.

## 6 Conclusion

Despite the limitations, this study suggests that creative, culturally relevant programs such as MONOZUKURI may be promising for enhancing executive function in community‐dwelling older adults. Learning creative activity skills may help prevent the decline of executive function, which is closely linked to daily functioning and quality of life. Further research with larger samples, longer follow‐up periods, and more rigorously matched control conditions will be essential to validate and expand on these findings. In particular, applying this program to older adults with cognitive decline may contribute to supporting their ability to live well with dementia.

## Author Contributions

**Ai Iizuka**: writing – original draft, formal analysis, project administration, visualization. **Susumu Ogawa**: investigation, writing – review and editing, data curation, formal analysis, software. **Daisuke Cho**: investigation, writing – review and editing, data curation, project administration. **Kyoko Fujihira**: investigation, writing – review and editing, data curation, project administration. **Yan Li**: investigation, writing – review and editing. **Kenichiro Sato**: investigation, writing – review and editing. **Keigo Hinakura**: investigation, writing – review and editing. **Kimi Estela Kobayashi‐Cuya**: investigation, writing – review and editing. **Hiroyuki Suzuki**: conceptualization, funding acquisition, writing – review and editing, supervision, project administration, methodology, validation, resources.

## Ethics Statement

Ethical clearance for this research was granted by the Institutional Review Board and Ethics Committee of the Tokyo Metropolitan Institute for Geriatrics and Gerontology in July 2020. The study adhered to the principles outlined in the Declaration of Helsinki.

## Consent

Prior to participation, all individuals were informed about the study's objectives, procedures, and ethical considerations. Written informed consent was obtained from each participant in accordance with the principles outlined in the Declaration of Helsinki.

## Conflicts of Interest

The authors declare no conflicts of interest.

## Peer Review

The peer review history for this article is available at https://publons.com/publon/10.1002/brb3.70888.

## Supporting information




**Supplementary Materials**: brb370888‐sup‐0001‐SuppMat.pdf

## Data Availability

The datasets generated and analyzed during the present study are available from the corresponding author upon reasonable request.
