# Supplementary material for: The Effects of the MONOZUKURI Program on Executive Function Among Community‐Dwelling Older Adults: A Randomized Controlled Trial
Source: Brain Behav. 2025 Sep 27;15(10):e70888. doi: 10.1002/brb3.70888 (PMC12475996; doi:10.1002/brb3.70888)
Supplement: Supplementary file 1 — Supplementary Materials: brb370888‐sup‐0001‐SuppMat.pdf [file BRB3-15-e70888-s001.pdf]

Supplementary 1. Content of the MONOZUKURI program.

Making accessories		
1st	Twisted knot pearl bracelets	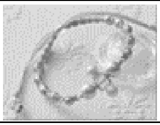
2nd	Myoga knot pendants	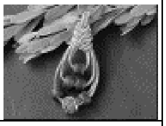
3rd	Color-changing bracelets	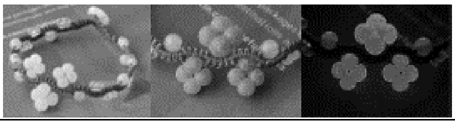
4th	Framing pendants	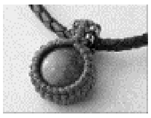
5th	Camellia brooches with Awaji-knot	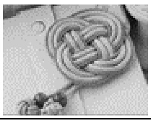
6th	Learning how to wear the handcrafted accessories and taking pictures	
Decorative sushi		
7th	Sakura and rabbit designs	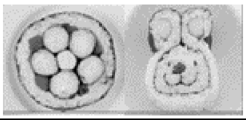
8th	Rose and panda designs	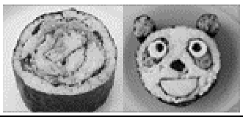
9th	A four-way roll designs	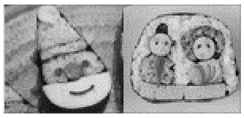
10th	Pine, bamboo, plum fan and bear designs	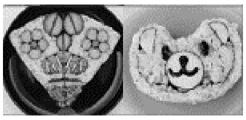
11th	Snail and original designs	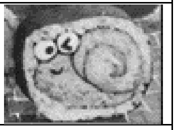
12th	Collaborative designs by all participants	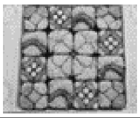
